# Exploring Older Adults’ Willingness to Install Home Surveil-Lance Systems in Taiwan: Factors and Privacy Concerns

**DOI:** 10.3390/healthcare11111616

**Published:** 2023-06-01

**Authors:** Chang-Yueh Wang, Fang-Suey Lin

**Affiliations:** Graduate School of Design, National Yunlin University of Science and Technology, Yunlin 64002, Taiwan; linfs@yuntech.edu.tw

**Keywords:** aging in place, home surveillance systems, privacy concerns, image privacy protection, technology acceptance

## Abstract

Taiwan has a rapidly increasing aging population with a considerably high life expectancy rate, which poses challenges for healthcare and medical systems. This study examines three key factors: safety concerns, family expectations, and privacy concerns, and their influence on surveillance system installation decisions. A cross-sectional study was conducted involving a group of physically active older adults in Taiwan, using a questionnaire to collect data on the reasons for in-stalling a surveillance system and preferences for three image privacy protection techniques: blurring the face and transformation to a 2D or 3D character. The study concluded that while safety concerns and family expectations facilitate the adoption of surveillance systems, privacy concerns serve as a significant barrier. Furthermore, older adults showed a clear preference for avatar-based privacy protection methods over simpler techniques, such as blurring. The outcomes of this research will be instrumental in shaping the development of privacy-conscious home surveillance technologies, adeptly balancing safety and privacy. This understanding can pave the way for technology design that skillfully balances privacy concerns with remote monitoring quality, thereby enhancing the well-being and safety of this demographic. These results could possibly be extended to other demographics as well.

## 1. Introduction

The global population of people aged 65 or older is expected to rise from 10% in 2022 to 16% in 2050 [1], mainly due to improved survival rates and decreased fertility levels. The aging of the population is an even more concerning issue in east Asian countries such as Japan, South Korea, Singapore, and Taiwan [2]. The population of older adults (over 65 years old) has reached a new high of 17.68% as of 2023 [3]. Over 57% of individuals aged 65 years or above in Taiwan had a consistent exercise routine of over 150 min per week [4], which indicates that the majority of the senior population in Taiwan is physically active. However, at the same time, a recent report also shows that 84.7% of the older adults are suffering from at least one chronic disease [5]. In Taiwan, while the healthcare system covers the majority of older adults and provides timely medical care, there is a growing demand for assisted ambient living systems as the families of older adults become more concerned about their well-being. Additionally, the Taiwanese government plans to use technological aids to assist older adults as a means of further improving their quality of life and well-being.

Aging in place is a complex issue, and older individuals value their connections to their homes and broader community, including social networks, familiarity, and resources, as highlighted in [6]. The study also emphasizes that there is no one-size-fits-all solution for an ideal living environment as seniors age in place, due to varying preferences and requirements. In a similar context, Taiwan’s rapidly aging society faces several challenges, including the need for age-friendly environments and intergenerational cohesion [7]. As traditional family caregiving declines and more older adults live separately from their families, fostering community-based care and aging in place becomes crucial. While the government is working to promote age-friendly cities, developing culturally appropriate solutions that prioritize aging in place is essential to address the overall needs of Taiwan’s elderly population. A systematic review presented in [8], indicated that the factors influencing the acceptance of technology for aging in place among community-dwelling older adults include concerns regarding technology, expected benefits, need for technology, alternatives, social influence, and personal characteristics. Additionally, the study suggested that in order to promote acceptance and support aging in place, practitioners should address individual concerns, communicate concrete benefits, involve supportive individuals, and be sensitive to alternative solutions.

In recent years, camera-based surveillance systems have emerged as a potential solution to assist older adults aging in place. These systems are designed to provide remote monitoring, fall detection, activity monitoring, and other safety features for older individuals, whether they are living alone or with family. By ensuring the well-being of older individuals while they are home alone, these systems can promptly alert emergency services or caregivers in the event of an emergency. This method of supporting aging in place helps address the challenges faced by an aging population while promoting independence and peace of mind for older adults and their families. In [9], a low-cost fall detection system for smart homes using image processing technique is presented that achieves over 96% accuracy in detecting falls in controlled environments. An omni-directional vision sensor-based remote monitoring system for elderly persons has been proposed in [10], enabling real-time health care through motion object tracking, human posture recognition, and behavior analysis, achieving high monitoring accuracy. In [11], a computer vision-based system using a stereo depth camera was developed to monitor the actions of the elderly, enabling real-time action detection and recognition with potential applications in active and assisted living, anomaly detection, sleep quality analysis, and emergency alerting systems. Another vision-based monitoring system is introduced in [12], using Hidden Markov Model to differentiate falls from normal states in elderly people. In [13], a vision-based system was developed that achieves high accuracy in recognizing poses and actions while integrating an end-user application that allowed guardians to efficiently explore events related to the monitored individual. A real-time monitoring prediction system for early detection of high fall risk in hospitalized elderly patients is developed in [14]. In a systematic review presented in [15], the effects of nocturnal digital surveillance technologies on health, welfare, and social care provision outcomes for individuals aged 50 years and older are examined in comparison to standard care. While this study concluded that there is no significant difference between the quality of monitoring between standard or nocturnal cameras, it still points out that overall, a monitoring system is effective at delivering alerts in the event of an emergency. All these systems, along with many other commercial solutions, rely on camera-based surveillance and often incorporate image processing techniques and artificial intelligence to improve overall accuracy. Some also consider the energy efficiency of video surveillance systems, as discussed in [16,17].

While many of the recent technological advancements in elderly monitoring systems are mostly focused on increasing accuracy and efficiency, factors such as privacy are usually ignored during development stages. Ambient intelligence in healthcare, involving contactless sensors and wearable devices coupled with machine learning algorithms, offers potential improvements in care quality but presents ethical challenges, including privacy, data management, and informed consent [18], which must also be addressed for widespread acceptance [19]. A study conducted among older adults from the United States and Canada [20] pointed out that the ambient assisted living (AAL) technologies can improve security and self-confidence for seniors, but they may also alter the perceptions of the home and give rise to ethical concerns about privacy and the social construction of dependences. Therefore, ensuring a balance between perceived usefulness, ease of use, and privacy concerns [21,22] is required for widespread acceptance of monitoring systems for older adults. One way of addressing such concerns includes involving the older adults during the development stage of such monitoring systems [23].

Concerns about privacy in surveillance technologies for elderly care need to be addressed. These technologies may unintentionally reproduce social inequalities, reinforce ageism, and limit personal freedom [24]. It is important to carefully examine their design and use within the broader context of political, economic, and social factors affecting aging populations. At the same time, some older individuals may see electronic care monitoring as a way to stay independent at home; however, the importance of personal choices still remains the dominant factor when choosing surveillance systems [25]. Several studies show that perceived usefulness is a key factor in older adults’ attitudes and willingness to use smart home products due to their safety benefits [26,27,28,29,30]. However, affordability, technology skepticism, privacy concerns, and return on investment also significantly impact older adults’ intentions to adopt these technologies.

Several new developments in the area of elderly surveillance are addressing the privacy concerns of the older adults. As presented in [31,32], vision-based systems are being developed where only skeletal data obtained after post processing the raw video feed are used to generate the actions and events data of those being monitored. In recent years, environmental sensors and wearables are also gaining popularity to assist the elderly in living independently and minimally obstructing their privacy and data security [33,34,35,36]. In recent developments, as presented in [37], the research outlines the challenges in adopting assisted living technologies among the aging population and proposes a holistic solution that integrates these technologies into a mobile robotic platform for in-home assistance. Although these technologies offer data security and privacy protection for seniors, their complexity and potential high costs can deter adoption. As a result, video surveillance, a widely used and understood method for monitoring activities and emergency events, requires particular focus on preserving privacy.

In this research, two innovative and one traditional method for image privacy protection method are surveyed for their potential to improve the acceptance of video surveillance among healthy older adults. The study’s focus on healthy older adults was largely influenced by two key considerations. Firstly, healthy older adults, generally capable of living independently, provided a unique perspective for understanding their willingness and preferences towards home surveillance systems, anticipating future needs. These individuals often spend considerable periods alone, which raises concerns among family members during such intervals. Secondly, this focus allowed the exploration of the decision-making processes of these older adults while their health status still permitted a significant degree of independence. This paper presents a cross-sectional study focusing on following points:

- This study explores the factors influencing the willingness of healthy older adults in Taiwan to install privacy-preserved home surveillance systems.
- This study analyzes the survey data to find the seniors’ preferences regarding the level of privacy protection.

## 2. Materials and Methods

### 2.1. Research Context

**Figure 1:**
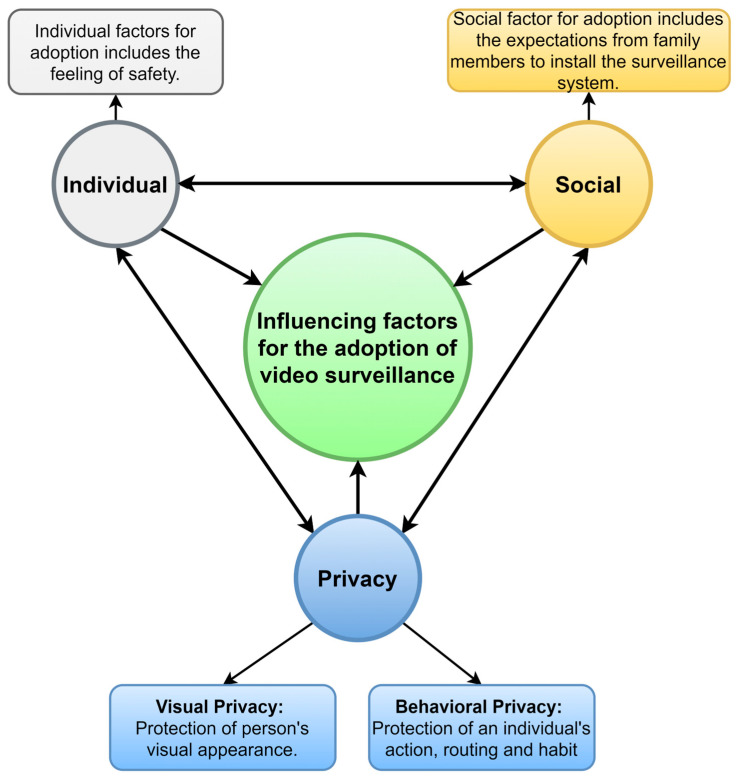
Research framework for analyzing the relationship between adoption of video surveillance and its influencing factors, including individual, social, and privacy factors.

This study aims to explore the preferences and factors that influence the willingness of healthy older adults in Taiwan to adopt home surveillance systems with privacy preservation features, as well as to understand their desired level of privacy protection. Building on previous studies on acceptance of video surveillance system [18,19,20,21,22,23], this study extracts three main factors with which to analyze the willingness to install a surveillance system within the context of Taiwan’s elderly demography: individual factors, social factors, and privacy factors. This study develops the framework for adoption of video surveillance by older adults in Taiwan, as shown in Figure 1. Individual factors include perceived usefulness, which is a feeling of safety/security (referred as “For Safety” here on) in this case. The social factor is interpreted as the expectations from family members to install a monitoring system (referred as “Family Expectation” from here on). Family’s expectations for their senior parents’ security and wellbeing are a major factor for the acceptance of home surveillance. This study separates another major factor, the privacy factor, into two main attributes: “Visual Privacy” and “Behavioral Privacy”. The two types of privacy can be described as follows:

- Visual Privacy: This can be described as the protection of a person’s visual appearance, including their face, body, and any identifiable features. In the context of video surveillance, it can be addressed by using techniques such as blurring or pixelating faces [38] or replacing the whole body images with avatars to ensure the person’s privacy. Recent advancements in the machine learning approach to image and video processing has shown many techniques [39,40,41] for converting human images in videos to two-dimensional (2D) or three-dimensional (3D) avatars.
- Behavioral Privacy: this type of privacy pertains to the protection of an individual’s actions, routines, and habits. In the context of video surveillance, behavioral privacy can be protected by ensuring that only relevant data are collected, by limiting the data history, or by using secure data processing techniques. In practice, ref. [42] shows context-aware machine learning models can be used to analyze the video data to extract the relevant information about the scene. A smart surveillance camera system [43] can be also used to process the scene data locally and transmit only emergency and critical events, protecting the overall behavioral privacy of the person.

Using the above framework, this study aims to address three research questions: (1) what factors, specifically individual, social, and privacy factors, influence the willingness of healthy older adults in Taiwan to install home surveillance systems? (2) What is the desired level of privacy expectation among the healthy older adults in Taiwan when using home surveillance systems, especially in terms of visual or behavioral privacy? (3) What are preferences of healthy older adults in Taiwan regarding home surveillance systems with privacy protection features? By addressing the above research questions, this study provides valuable insights through survey data analysis for future research, policy, and product design/development for privacy-protected video surveillance system for older adults in Taiwan.

### 2.2. Data Collection

This research uses a cross-sectional study method where data were collected through a structured questionnaire designed to explore the preferences and factors that influence the willingness of healthy older adults in Taiwan to install home surveillance systems with or without privacy preservation features. Using the previous studies mentioned in Section 1 and Section 2.1, the questionnaire also directly addresses whether older adults are more concerned about their visual privacy, their behavioral privacy, or both. The target population for this study was older adults aged above 65 years old who identified themselves as healthy and physically active. A total of 50 healthy older adults were given the questionnaire out of 87 older adults that were approached during the data collection phase. To address the technical aspects of the questionnaire used in this study and ensure accurate comprehension of the research context and questions, a detailed explanation of participants was provided prior to data collection. Informed consent was also obtained from all participants to determine whether they fully understood the scope of study and were willing to participate based on explanation of the study objectives. This approach was used to improve participants’ understanding of the study’s objectives and clarify any potential misconceptions or ambiguities, thus promoting the validity of the collected data.

**Figure 2:**
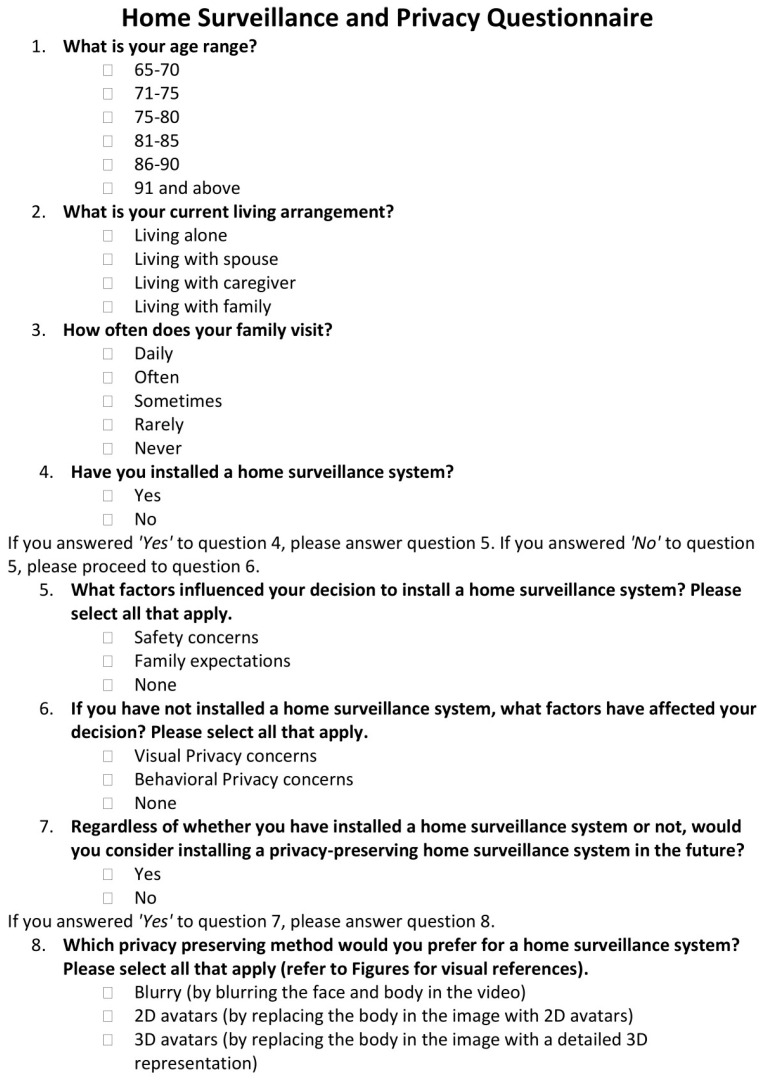
English version of questionnaire used in survey.

**Figure 3:**
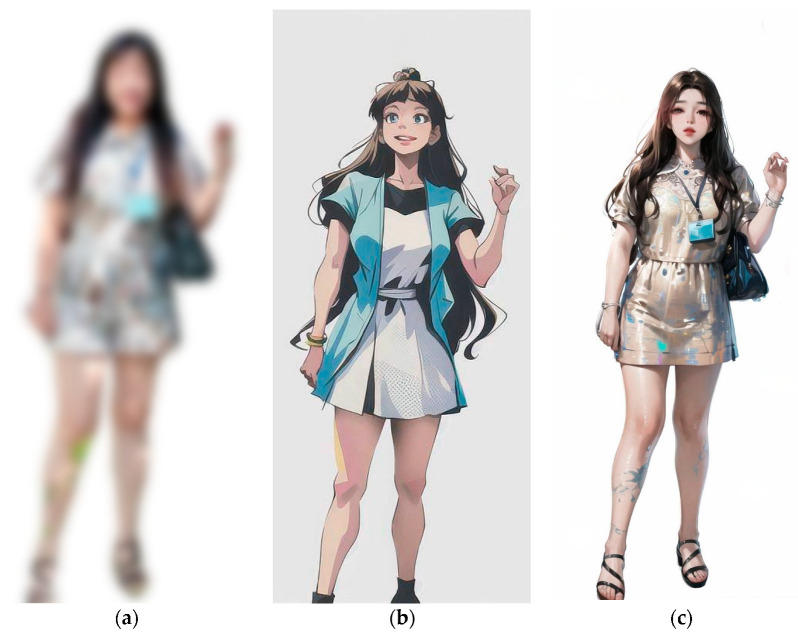
Visual illustration of privacy protection methods used in survey (**a**) Blurry, (**b**) 2D avatar, and (**c**) 3D avatar.

For clarity and ease of understanding for the older adult participants, the study adopted a Yes/No questionnaire format, which is less complicated than the Likert scale. The questionnaire, as shown in Figure 2, included yes/no type and multiple-choice questions to identify whether the participants had already installed a home-surveillance system. For the individuals who answered “yes”, further multiple-choice question was presented to determine which factor influenced the installation of surveillance system as “Safety”, “Family Expectation” or both. For participants who answered “no”, the multiple-choice question proceeded to identify the factors that affected their decision as “Visual Privacy”, “Behavioral Privacy”, both, or none. Another set of questions was also asked of both groups of participants who answered “yes” or “no” for the first question: whether they intend to install a privacy preserving home surveillance system. For those who answered “yes”, the questionnaire provided a multiple-choice question offering several privacy-preserving methods to choose from: blurry (blurring the face and body in the video) as shown in Figure 3a, 2D avatars (replacing the body in image with 2D avatars) as shown in Figure 3b, and/or 3D avatar (replacing the body in image with a detailed 3D representation), as shown in Figure 3c. The questionnaire also comprises questions regarding the age range (a 5-year scale starting from 65 years old) and living arrangements, i.e., whether they live alone, with a spouse, with a caregiver, or with their family. Family visit frequency was categorized in 5 levels (Daily, Often, Sometimes, Rarely and Never). 

The questionnaire shown in Figure 2 directly addresses three research questions formulated in Section 2.1:

1. Factors influencing the willingness of older adults in Taiwan to install home surveillance systems are explored through Questions 4, 5, and 6. Question 4 initially determines if a home surveillance system has been installed, and if so, Question 5 further probes the motivating factors behind this decision (e.g., safety concerns, family expectations). Alternatively, if a system has not been installed, Question 6 delves into the concerns or obstacles preventing such installation.
2. The desired level of privacy expectation among older adults in Taiwan when using home surveillance systems is gauged through Questions 6 and 7. Question 6 investigates the privacy concerns of respondents who have not installed a surveillance system, while Question 7 identifies respondents’ openness to considering a privacy-preserving home surveillance system in the future.
3. The preferences of older adults regarding home surveillance systems with privacy protection features are explored in Question 8. This question assesses the preferred methods of privacy preservation for those respondents who expressed a willingness to consider a privacy-preserving system in the future (as indicated in their response to Question 7).

Finally, Questions 1–3 provide valuable context by gathering demographic data (age and living arrangements) and data about social interaction (frequency of family visits), aiding the interpretation of the responses to the primary research questions.

### 2.3. Data Analysis

This study uses the descriptive data analysis to perform the initial analysis of the dataset regarding the attributes such as age, gender, living conditions, and family visit frequency. Given the small size of the samples, this study employed a two-sided Fisher’s exact test [44] to determine the non-random association between variables using 2 × 2 contingency tables to answer the research questions planned in Section 2.1. The main reason for using Fisher’s exact test over the Chi-square test is because Fisher’s exact test does not require the expected cell counts to be greater than 5 for at least 80% of the cells. This makes it a suitable method when working with the smaller datasets [45,46] such as those presented in this study. This study also employs the phi coefficient (φ) to measure the direction and strength of association [47,48] between binary variables.

## 3. Results

Given the study’s focus on healthy older adults in Taiwan, accounting for the margin of error is an important consideration. The study adopted a random sampling method, and the target demographic—adults over 65 as of 2023 [3]—constitutes 4,122,171 individuals. With a 95% confidence interval and a z-score value of 1.96, this study yields a margin of error approximated at ±14% [49,50]. Despite a relatively high margin of error, the findings from this limited sample can still offer valuable preliminary insights for understanding the research objectives.

### 3.1. Sampling and Participant Characteristics

In this subsection, an overview of the participant’s characteristics is provided that will help contextualize the main findings. This subsection mainly presents descriptive statistics for the healthy older adults who took part in this study, including their age distribution, living arrangements, family visit frequency, and if they have already had a home surveillance system installed. There were 23 male participants (46%) out of a total 50 older adults who took the questionnaire. Table 1 presents the frequency table for the age distribution of participants, with most of the participants falling within the range of 65–70 and 71–75 years old. In Table 2, the living arrangement of the participants is displayed. Most of the participants are living with family or with their spouses, followed by participants who are living alone and living with their caregivers. The frequency of family visits is shown in Table 3, and it is clear from the table that most of the older adults have a daily visit from at least one of the family members. The percentage of participants who have already installed a surveillance system in their homes is only 32% compared to 68% of who have installed no surveillance systems, as shown in Table 4. This shows that the majority of the healthy older adults do not have any video surveillance systems installed in their home or living compartments. 

### 3.2. What Factors Influence the Willingness of Healthy Older Adults in Taiwan to Install Home Surveillance Systems?

As formulated in Section 2.1, we consider three major factors, including, “Safety”, “Family Expectation”, and “Privacy”, to be the influencing factors for installing or not installing a home surveillance system. We also categorized the “Privacy” factor into two types: “Visual Privacy” and “Behavioral Privacy”. It should be noted that the contingency table for “Visual Privacy” and “Behavioral Privacy” also includes the concerns raised by the participants who already have a surveillance system installed in their homes, while the table for “Privacy” only includes the data of the participants who do not have any surveillance system installed in their homes. In Table 5, cross tabulation of results is presented based on answers from the participants. 

The contingency table for cross-tabulation between installation of surveillance system and “Safety” factor showed a strong positive association (φ = 0.954, *p* < 0.001). Additionally, the Fisher’s exact test for safety consideration (*p* < 0.001) shows a significant relationship between a “Safety” factor and having a surveillance system. Out of 50 older adults, 34 did not install a home surveillance system and reported that safety was not an influencing factor. At the same time, 15 out of 16 participants who installed a surveillance system reported that safety concerns indeed influenced their decision to do so. Similarly, a significant association was found between installation of a home surveillance system and “Family Expectation” (*p* < 0.001) using Fisher’s exact test. The strength of association was positive and high (φ = 0.683, *p* < 0.001) in the symmetric measures analysis. The cross-tabulation also shows that 9 out of 16 participants have installed a home surveillance system because of the influence of their family members. 

When taking the “Privacy” factor into account, 34 out of 34 participants who did not install a surveillance system did so because they were concerned about their privacy. This shows a perfect negative association (φ = −1.0, *p* < 0.001) between privacy concerns and installation of a surveillance system, which is also confirmed by Fisher’s exact test (*p* < 0.001). When discussing the concerns about specific types of privacy in terms of “Visual Privacy”, the result of Fisher’s exact test shows a statistically significant association (*p* < 0.001) with the decision to install a surveillance system. The symmetric measures analysis using the Phi coefficient also shows a high negative association (φ = −0.806, *p* < 0.001) between these two variables, as 29 out of 34 participants who did not install a surveillance system reported that they did so because they were concerned about their visual privacy. For the “Behavioral Privacy”, based the on results of the analysis, Fisher’s exact test shows strong association (*p* < 0.001) between “Behavioral Privacy” and installation of a surveillance system. There is also a significant negative association (φ = −0.806, *p* < 0.001) between installation of a surveillance system and concerns for behavioral privacy by the participants. Although the results show that “Visual Privacy” is a more prominent factor than “Behavioral Privacy,” it is evident that privacy concerns, in general, have a substantial influence on installation of a surveillance system by older adults. Based on this analysis, the factors can be ranked by their strength of association (φ value), with “Privacy” concerns being the most powerful deterrent for non-installation, while “Safety” emerges as the primary motivator for those who choose to install a surveillance system, followed by “Family Expectation.”

### 3.3. What Is the Desired Level of Privacy Expectation among the Healthy Older Adults in Taiwan When Using Home Surveillance Systems, Especially in Terms of Visual or Behavioral Privacy?

In order to understand the desired level of privacy expectation among healthy older adults while using home surveillance systems, it important to understand which type of privacy is important to the participants of this study, and whether in fact there is an association between visual and behavioral privacy. In Table 6, the contingency table between “Visual Privacy” concerns and “Behavioral Privacy” concerns is shown. Based on Fisher’s exact test, it can be concluded there is indeed a statistically significant association (*p* = 0.042) between two variables. Using the odds ratio measure, it can be reported that the “Visual Privacy” concern is 3.94 times higher among individuals who also have “Behavioral Privacy” concerns. In symmetric measures analysis (φ = 0.313, *p* = 0.053), a moderate positive association can be observed. However, the *p*-value above 0.05 may deem this association insignificant.

Table 7 shows cross-tabulation of responses from participants regarding whether they will install a privacy-preserved version of the surveillance system regarding their concerns for a specific type of privacy. This table was constructed to analyze whether privacy concerns have a non-random association with the willingness to install a privacy-preserved version of the home surveillance system.

The analysis of Table 7 shows there is no significant association between willingness to install a privacy preserving surveillance system and “Visual Privacy” (*p* = 1.0, Fisher’s Exact Test) or “Behavioral Privacy” (*p* = 1.0, Fisher’s Exact Test) concerns of the participants. While Fisher’s exact test reported no statistical significance for both types of privacy concerns and willingness to install a privacy protected surveillance system, this study found that the majority of the participants (n = 41, 82%) prefer a privacy-protected version of a home surveillance system, regardless of their privacy concerns.

### 3.4. What Are the Preferences of Healthy Older Adults in Taiwan Regarding Home Surveillance Systems with Privacy Protection Features?

In this study’s questionnaire, participants were presented with three distinct privacy protection methods to choose from. These methods involve altering the individual’s image within the surveillance video by transforming it into a “2D Avatar,” a “3D Avatar,” or by “Blurring” the image. This selection allows respondents to express their preferred approach to maintaining privacy in the context of surveillance systems. Table 8 presents the contingency table illustrating the distribution of these methods in relation to the participants’ inclination towards the installation of privacy preserving surveillance systems.

Using Fisher’s exact test, a statistically significant association (*p* < 0.001) between converting images into “2D Avatar” and willingness to install the privacy-protected system was found. There is also a high positive association (φ = 0.683, *p* < 0.001) for participants choosing the “2D Avatar” method if they also choose to install a privacy-preserving system. Similarly, the “3D Avatar” method also shows a significant association (*p* = 0.02, Fisher’s exact test), but only moderate positive association (φ = 0.336, *p* = 0.047) regarding willingness to install a privacy-preserving system. However, no statistically significant association (*p* = 0.57, Fisher’s exact test) was observed for the “Blurring” method and willingness to install the privacy-preserving system. Overall, observations showed that 82.9% of older adults who are willing to install a privacy-protected surveillance system prefer the “2D Avatar” method, while 41.4% also prefer the “3D Avatar” method, but only 12% prefer the “Blurring method” of privacy protection.

## 4. Discussion

The respondent characteristics show a diverse range of ages, living arrangements, and family visit frequencies, which are essential to understand the context of the study and interpret the results. It has been established several studies prior to this on the topic of technology adoption that older adults have unique needs and concerns about their privacy, and these attributes can help explain the factors affecting their decision-making process in context of home surveillance system. While privacy concerns are the main focus of this study, it is also important to analyze what other factors, such as individual or social factors, affect the decision of older adults.

The results of this study demonstrated that safety (individual factor), family expectation (social factor), and privacy concerns (privacy factor) play important roles in the willingness of older adults to install home surveillance systems. The prominence of safety as a motivator, with 15 out of 16 participants (93.75%) who installed a surveillance system citing safety concerns, aligns with the prior research that emphasizes the importance of individual factors and sense of security in one’s living environment. This factor, as an individual factor that affects the decision, is especially important because older adults are more vulnerable to various risks (e.g., falling, emergency medical conditions, etc.) while staying alone in their places. In this study, the second motivator for installing a surveillance system is social pressure or family expectations. This highlights that the influence of family members on their older parent’s decision to install a monitoring system is evident, as the cross-tabulation shows that 9 out of 16 participants (56.25%) installed the surveillance system due to influence from their family members.

Privacy concerns of older adults in Taiwan were found to be a dominant deterrent against installing a home surveillance system, with 34 out of 34 participants who did not install a surveillance system citing privacy concerns. Among this group, 24 out of 34 participants (85.29%) were concerned about their visual privacy, while 21 out of 34 participants (61.76%) were also concerned about their behavioral privacy. This finding highlights the significance of privacy considerations in the context of technology use among older adults in Taiwan, as the expectation of privacy is very important for maintaining dignity and autonomy. The strong association between privacy concerns and non-installation of surveillance system suggests alignment with the previous studies on the same matter in other demographic regions and proves that in the context of older adults in Taiwan, the same theory can be applied.

Regarding the expectation of privacy, our study found a significant association between visual and behavioral privacy concerns. This result also shows that while healthy older adults in Taiwan value both aspects of privacy, the expectation of visual privacy is more dominant than that of behavioral privacy. This study also showed that older adults in this population preferred a privacy-protected version of a home surveillance system regardless of their concerns for visual or behavioral privacy, with 82% participants expressing interest. This further emphasized the importance of privacy protection for this population.

Further, this study also explored the privacy protection features based on image privacy protection methods. The results revealed that a substantial majority (82.9%) of the participants favored the “2D Avatar” method among the group of participants who were willing to install a privacy-protected system. At the same time, 41.4% participants also favored the “3D Avatar” method of image privacy protection, while the image blurring method was found to be the least favorite. This finding suggests that healthy older adults prefer methods that transform their images within the surveillance video over simply blurring their image. The preference of avatar-based method could be attributed to these methods offering a higher level of privacy protection by completely replacing the original image while retaining the functionality of the surveillance system. This also suggests that future developments in the area of privacy-protected surveillance systems in the Taiwan region can focus on developing systems that replace the original images with avatars rather than making the images unidentifiable by blurring or wrapping them. 

In summary, this study contributes to the understanding of factors influencing the willingness of healthy older adults in Taiwan to install a home surveillance system and their privacy preferences. The results emphasize the importance of individual, social and, most dominantly, privacy concerns. Incorporating feedback from older adults and addressing privacy concerns in the design of home surveillance systems can significantly enhance their acceptance among Taiwan’s population. This, in turn, can contribute to the promotion of the overall well-being and safety of older adults while also ensuring the protection of their privacy rights.

## 5. Conclusions

This study has provided valuable insights into the factors influencing the installation of home surveillance systems among healthy older adults in Taiwan. This study concludes that privacy concerns are a major deterrent factor for adoption of surveillance systems, while concerns of safety and family expectations play a positive role in their adoption. Furthermore, the results also emphasize the importance of visual and behavioral privacy protection for this population, with a clear preference for avatar-based methods over simpler techniques, such as blurring. 

These insights have significant implications for the development of privacy-protected home surveillance systems tailored to the needs of healthy older adults in Taiwan. It is crucial to understand that the focus on healthy older adults is intentional, as they may not currently have immediate health concerns necessitating the installation of a monitoring system. However, they typically spend significant time alone at home without the company of their family members. Therefore, this study aims to evaluate the trade-offs between the perceived benefits of home surveillance and potential privacy implications. A privacy-protected system can address the safety concerns and family expectations while prioritizing the privacy concerns of users as well. The outcomes of this study can facilitate the development of video surveillance technologies that promote the well-being and safety of healthy older adults. 

One notable limitation of this study is the small sample size, which could limit the generalizability of the findings. Notably, the adopted sample size yields a margin of error approximated at ±14%, potentially impacting the precision of our estimates. Despite this, the findings from the sample offer valuable preliminary insights that can be informative for understanding the research objectives of this study. The sample primarily comprises older adults from a region in Taiwan, raising potential difficulties in generalizing the findings to other countries, cultural backgrounds, or age groups. In line with existing research and models such as the Technology Acceptance Model (TAM) and the unified theory of acceptance and use of technology (UTAUT) model, the three key factors explored in this study—privacy concerns, safety concerns, and family expectations—may not be exclusive to the older adults, suggesting the possibility that our findings could apply to younger adults. However, without targeted research within these different demographic groups, we caution against overgeneralization and encourage research in this direction for more clarity.

These limitations open up future research avenues to extend the findings to a broader population and different age groups. Longitudinal study designs could help us to further understand how preferences and attitudes towards home surveillance systems evolve over time and in response to using privacy-protected systems. Exploring diverse geographical and cultural contexts would provide a more comprehensive understanding of the adoption factors for home surveillance systems, particularly in light of recent technological advancements.

While the study focuses on the Taiwan demographic, the findings may have broader implications and could provide valuable insights into the development of video-based assisted living solutions worldwide. Future studies could build upon these findings and technological aspects to develop technologies that better meet the unique needs and preferences of various populations.

## Figures and Tables

**Table 1:** Frequency distribution table of age of the participants.

Age Range	Frequency	Percent
65–70	16	32.0
71–75	15	30.0
76–80	4	8.0
81–85	7	14.0
86–90	5	10.0
>91	3	6.0
Total	50	100.0

**Table 2:** Frequency distribution table of living arrangement of the participants.

Living Arrangement	Frequency	Percent
Alone	11	22.0
With Spouse	17	34.0
With Family	19	38.0
With Caregiver	3	6.0
Total	50	100.0

**Table 3:** Frequency distribution table of family visits to the participants.

Living Arrangement	Frequency	Percent
Never	2	4.0
Rarely	4	8.0
Sometimes	7	14.0
Often	5	10.0
Daily	32	64.0
Total	50	100.0

**Table 4:** Frequency distribution table of participants with or without a video surveillance system installed in their homes.

Surveillance Installed	Frequency	Percent
Yes	16	32.0
No	34	68.0
Total	50	100.0

**Table 5:** Cross-tabulation of binary variables examining the factors influencing the installation of surveillance systems among older adults.

		Safety		Family Expectation		Privacy		Visual Privacy		Behavioral Privacy	
		No	Yes	No	Yes	No	Yes	No	Yes	No	Yes
**Surveillance** **installed**	No	34	0	34	0	0	34	5	29	13	21
	Yes	1	15	7	9	16	0	16	0	16	0

**Table 6:** Cross-tabulation of binary variables examining the visual and behavioral privacy factors.

		Behavioral Privacy	
		No	Yes
**Visual Privacy**	No	16	5
	Yes	13	16

**Table 7:** Cross-tabulation of binary variables examining the factors influencing the willingness to install a privacy-preserved surveillance system among older adults.

		Visual Privacy		Behavioral Privacy	
		No	Yes	No	Yes
**Will install privacy-preserved** **surveillance**	No	4	5	6	3
	Yes	17	24	23	18

**Table 8:** Cross-tabulation of binary variables examining the preferences for privacy preservation method and the willingness of installing a privacy-preserved surveillance system among older adults.

		2D Avatar		3D Avatar		Blurring	
		No	Yes	No	Yes	No	Yes
**Will install privacy-preserved** **surveillance**	No	9	0	9	0	9	0
	Yes	7	34	24	17	36	5

## Data Availability

The original survey data used by this study contain information that may include personal and sensitive information of the participants. To protect the privacy of the participants, a curated version of the dataset is available upon request. Interested parties may contact the corresponding author for access to the curated dataset.

## References

[1] Department of Economic and Social Affairs (2022). World Population Prospects 2022.

[2] Department of Economic and Social Affairs (2019). World Population Ageing 2019.

[3] Ministry of the Interior Household Registration Statistics Data Analysis in Feb 2023. https://www.ris.gov.tw/app/en/2121?sn=23069527.

[4] Ministry of Health and Welfare 2018 Health Promotion Administration Annual Report. https://www.hpa.gov.tw/EngPages/Detail.aspx?nodeid=1070&pid=11671.

[5] Ministry of Health and Welfare 2022 Health Promotion Administration Annual Report. https://www.hpa.gov.tw/EngPages/Detail.aspx?nodeid=1070&pid=16384.

[6] Wiles J.L., Leibing A., Guberman N., Reeve J., Allen R.E.S. (2012). The meaning of “aging in place” to older people. Gerontologist.

[7] Lin Y.Y., Huang C.S. (2016). Aging in Taiwan: Building a society for active aging and aging in place. Gerontologist.

[8] Peek S.T.M., Wouters E.J.M., van Hoof J., Luijkx K.G., Boeije H.R., Vrijhoef H.J.M. (2014). Factors influencing acceptance of technology for aging in place: A systematic review. Int. J. Med. Inform..

[9] De Miguel K., Brunete A., Hernando M., Gambao E. (2017). Home camera-based fall detection system for the elderly. Sensors.

[10] Xiang Y., Tang Y.P., Ma B.Q., Yan H.C., Jiang J., Tian X.Y. (2015). Remote safety monitoring for elderly persons based on omni-vision analysis. PLoS ONE.

[11] Zin T.T., Htet Y., Akagi Y., Tamura H., Kondo K., Araki S., Chosa E. (2021). Real-Time Action Recognition System for Elderly People Using Stereo Depth Camera. Sensors.

[12] Htun S.N.N., Zin T.T., Tin P. (2020). Image Processing Technique and Hidden Markov Model for an Elderly Care Monitoring System. J. Imaging.

[13] Buzzelli M., Albé A., Ciocca G. (2020). A Vision-Based System for Monitoring Elderly People at Home. Appl. Sci..

[14] Alharbi A.H., Hosni Mahmoud H.A. (2022). Intelligent Monitoring Model for Fall Risks of Hospitalized Elderly Patients. Healthcare.

[15] Richardson M.X., Ehn M., Stridsberg S.L., Redekop K., Wamala-Andersson S. (2021). Nocturnal digital surveillance in aged populations and its effects on health, welfare and social care provision: A systematic review. BMC Health Serv. Res..

[16] Liu L., Hou Y., He J., Lungu J., Dong R. (2020). An energy-efficient fall detection method based on FD-DNN for elderly people. Sensors (Switzerland).

[17] Oikonomou K.M., Kansizoglou I., Manaveli P., Grekidis A., Menychtas D., Aggelousis N., Sirakoulis G.C., Gasteratos A. Joint-Aware Action Recognition for Ambient Assisted Living. Proceedings of the IST 2022—IEEE International Conference on Imaging Systems and Techniques.

[18] Martinez-Martin N., Luo Z., Kaushal A., Adeli E., Haque A., Kelly S.S., Wieten S., Cho M.K., Magnus D., Fei-Fei L. (2021). Ethical issues in using ambient intelligence in health-care settings. Lancet Digit. Health.

[19] Gerke S., Yeung S., Cohen I.G. (2020). Ethical and Legal Aspects of Ambient Intelligence in Hospitals. JAMA.

[20] Mortenson W.B., Sixsmith A., Beringer R. (2016). No Place Like Home? Surveillance and What Home Means in Old Age. Can. J. Aging.

[21] Chen Z., Qi H., Wang L. (2021). Study on the types of elderly intelligent health management technology and the influencing factors of its adoption. Healthcare.

[22] Pirzada P., Wilde A., Doherty G.H., Harris-Birtill D. (2022). Ethics and acceptance of smart homes for older adults. Informatics Health Soc. Care.

[23] Ziefle M., Wilkowska W. Technology acceptability for medical assistance. Proceedings of the 2010 4th International Conference on Pervasive Computing Technologies for Healthcare.

[24] Kenner A.M. (2008). Securing the elderly body: Dementia, surveillance, and the politics of “aging in place”. Surveill. Soc..

[25] Essén A. (2008). The two facets of electronic care surveillance: An exploration of the views of older people who live with monitoring devices. Soc. Sci. Med..

[26] Tural E., Lu D., Austin Cole D. (2021). Safely and Actively Aging in Place: Older Adults’ Attitudes and Intentions Toward Smart Home Technologies. Gerontol. Geriatr. Med..

[27] Arthanat S., Wilcox J., Macuch M. (2019). Profiles and Predictors of Smart Home Technology Adoption by Older Adults. OTJR Occup. Particip. Health.

[28] Cao Y., Erdt M., Robert C., Naharudin N.B., Lee S.Q., Theng Y.L. (2022). Decision-making Factors Toward the Adoption of Smart Home Sensors by Older Adults in Singapore: Mixed Methods Study. JMIR Aging.

[29] Giger J.T., Pope N.D., Vogt H.B., Gutierrez C., Newland L.A., Lemke J., Lawler M.J. (2015). Remote patient monitoring acceptance trends among older adults residing in a frontier state. Comput. Human Behav..

[30] Jaschinski C., Allouch S.B., Peters O., Cachucho R., Van Dijk J.A.G.M. (2021). Acceptance of technologies for aging in place:a conceptual model. J. Med. Internet Res..

[31] Hbali Y., Hbali S., Ballihi L., Sadgal M. (2018). Skeleton-based human activity recognition for elderly monitoring systems. IET Comput. Vis..

[32] Sun H., Chen Y. (2022). Real-Time Elderly Monitoring for Senior Safety by Lightweight Human Action Recognition. Proceedings of the 2022 IEEE 16th International Symposium on Medical Information and Communication Technology (ISMICT).

[33] Gochoo M., Alnajjar F., Tan T.-H., Khalid S. (2021). Towards Privacy-Preserved Aging in Place: A Systematic Review. Sensors.

[34] Shao S., Yamamoto K., Kubota N. (2021). An elderly monitoring system based on multiple ultra-sensitive vibration and pneumatic sensors. J. Adv. Comput. Intell. Intell. Inform..

[35] Diraco G., Leone A., Siciliano P. (2017). A radar-based smart sensor for unobtrusive elderly monitoring in ambient assisted living applications. Biosensors.

[36] Issa M.E., Helm A.M., Al-Qaness M.A.A., Dahou A., Elaziz M.A., Damaševičius R. (2022). Human Activity Recognition Based on Embedded Sensor Data Fusion for the Internet of Healthcare Things. Healthcare.

[37] Keroglou C., Kansizoglou I., Michailidis P., Oikonomou K.M., Papapetros I.T., Dragkola P., Michailidis I.T., Gasteratos A., Kosmatopoulos E.B., Sirakoulis G.C. (2023). A Survey on Technical Challenges of Assistive Robotics for Elder People in Domestic Environments: The ASPiDA Concept. IEEE Trans. Med. Robot. Bionics.

[38] Padilla-López J.R., Chaaraoui A.A., Flórez-Revuelta F. (2015). Visual privacy protection methods: A survey. Expert Syst. Appl..

[39] Wu Y., Deng Y., Yang J., Wei F., Chen Q., Tong X. (2022). AniFaceGAN: Animatable 3D-Aware Face Image Generation for Video Avatars. arXiv.

[40] Li Z., Chen L., Liu C., Zhang F., Li Z., Gao Y., Ha Y., Xu C., Quan S., Xu Y. (2021). Animated 3D human avatars from a single image with GAN-based texture inference. Comput. Graph..

[41] Zhao H., Zhang J., Lai Y.K., Zheng Z., Xie Y., Liu Y., Li K. High-Fidelity Human Avatars from a Single RGB Camera. Proceedings of the IEEE/CVF Conference on Computer Vision and Pattern Recognition (CVPR).

[42] Brdiczka O., Crowley J.L., Reignier P. (2007). Learning Situation Models for Providing Context-Aware Services. Universal Access in Human-Computer Interaction.

[43] Fleck S., Straßer W. Towards secure and privacy sensitive surveillance. Proceedings of the Fourth ACM/IEEE International Conference on Distributed Smart Cameras.

[44] Fisher R.A. (1934). Statistical Methods for Research Workers.

[45] Agresti A. (2002). Categorical Data Analysis.

[46] Sprent P., Smeeton N.C. (2007). Applied Nonparametric Statistical Methods.

[47] Yule G.U. (1912). On the Methods of Measuring Association Between Two Attributes. J. R. Stat. Soc..

[48] Matthews B.W. (1975). Comparison of the predicted and observed secondary structure of T4 phage lysozyme. Biochim. Biophys. Acta—Protein Struct..

[49] Fowler F.J. (2014). Survey Research Methods.

[50] Isserlis L. (1918). On the Value of a Mean as Calculated from a Sample. J. R. Stat. Soc..

